# SlimShadey: A manipulation tool for multiple sequence alignments

**DOI:** 10.6026/97320630019659

**Published:** 2023-05-31

**Authors:** Avi Shah, Kodati Aneesha, Nayak Sudhir

**Affiliations:** 1 The College of New Jersey, 2000 Pennington Rd., Ewing, NJ, USA 08628-0718

**Keywords:** SlimShadey, manipulation, tool, multiple sequence alignments

## Abstract

The visualization of sequence alignments with the addition of meaningful shading and annotation is critical to convey the importance of structural elements, domains, motifs, and individual residues. Hence, we have developed a JavaFX based software package (SlimShadey) with an intuitive graphical user interface that allows for the creation and visualization of features on sequence alignments, as well as trimming and editing of subsequences. SlimShadey will run without modification on Windows 7 (or higher) and will also run on OS X / macOS, most Linux distributions, and servers. SlimShadey features real-time shading and comparison of residues based on user-defined measures of conservation such as frequency, user-selected substitution matrices, composition-based consensus sequence, regular expressions, and hidden Markov models. The software also allows users to generate custom sequence logos, configurable publication quality images of alignments with shading and annotation, and shareable self-contained project files for collaboration. SlimShadey is an open source freely available Java program. Compiled .jar executables, source code, supplementary materials including the user manual, links to video tutorials, and all sample data are available through the URLS at availability.

## Background:

There are several programs available for the analysis and visualization of multiple sequence alignments. For example, ALSCRIPT, ColorAlign, and MView were three of the earliest implementations for the shading of sequence alignments. ALSCRIPT relies on a command-line interface to shade and edit sequence alignments [1] and ColorAlign is optimized for simple greyscale shading [2]. MView is a web-based interface, currently hosted by EMBL-EBI, which shades in color but has limited functionality and is not customizable [3]. Other early implementations of Java-based shading or sequence alignments, such as JavaShade, are no longer supported [4]. More recently, the Jalviev and MEGA X (Molecular Evolutionary Genetics Analysis) suites allow for some manipulation of sequence alignments with a focus on sequence analysis [5- 6]. Notably, none of the currently available shading and visualization tools operate in real-time, i.e. to create a figure using new settings or parameters, a new command line or server request must be initiated.

**Figure 1:**
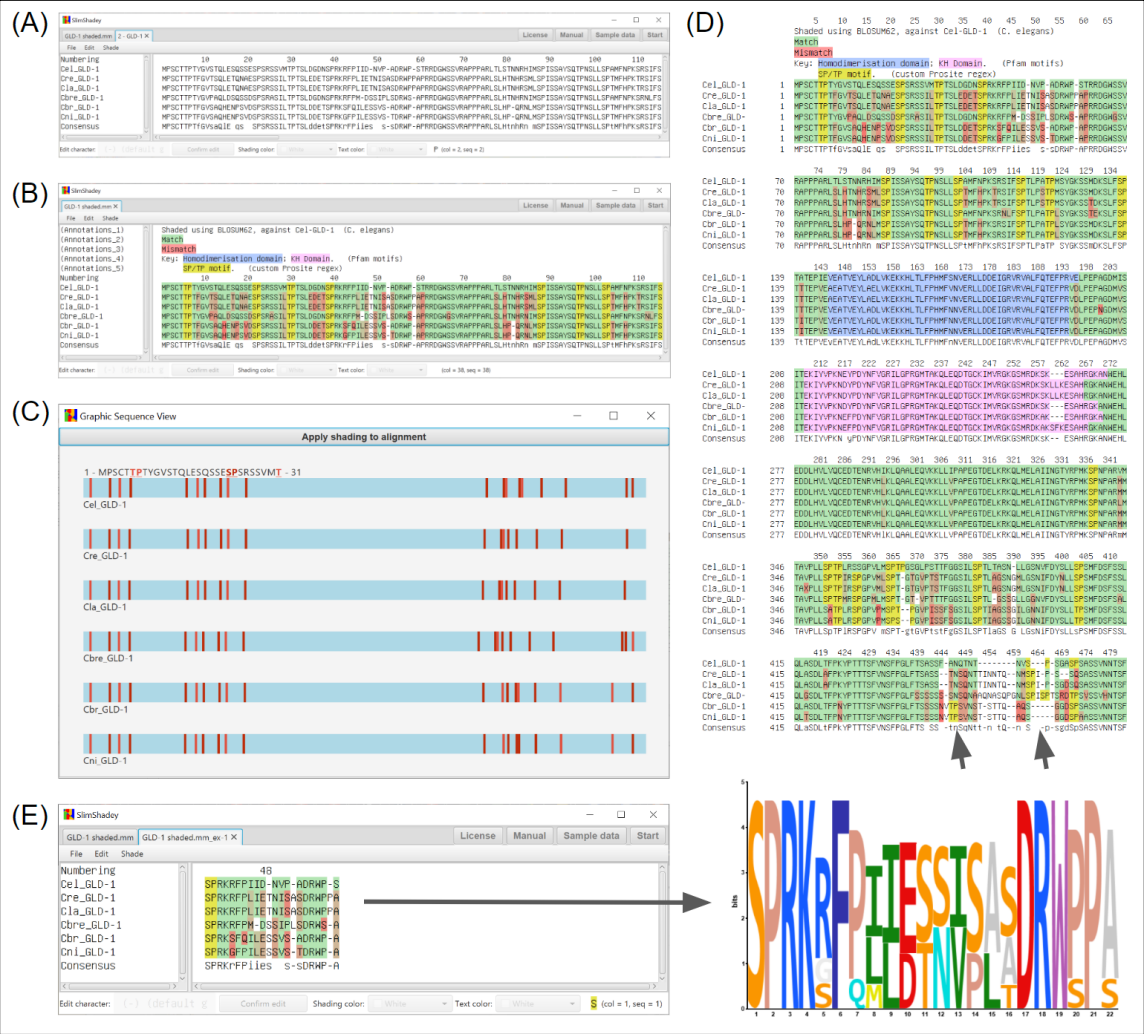
Processing of Caenorhabditis GLD-1 (defective in Germ Line Development) sequence alignment using SlimShadey. (A) Opal-aligned sequences homologous to Caenorhabditis elegans GLD-1 with >75% coverage obtained from WormBase release WS273 (https://wormbase.org/). (B) Shading of alignment from panel A. Base shading is BLOSUM62 gradient with green defined as a match to C. elegans and red defined as a mismatch to C. elegans, yellow is a custom regular expression highlighting (TP or SP, "[ST]-P"), Pfam (HMMER) defined KH domain (pink) and homodimerization domains (blue). (C) Bird's eye view of custom regular expression revealing clustering at N and C-terminal regions. (D) PNG output of complete alignment with all shading included. Arrows at the C-terminal end indicate the identification of motifs independent of position and the presence of gaps, respectively. (E) New tab (left) containing columns 39 to 60 of the original alignment, used to generate the sequence logo (right). Cel=C. elegans, Cre=C. remanei, Cla=C. latens, Cbre=C. brenneri, Cbr=C. briggsae, Cni=C. nigoni.

SlimShadey is a comprehensive and easy-to-use software package that allows for the real-time shading, coloring, editing, and production of sequence alignments, all contained in an intuitive graphical user interface (GUI, Figure 1). The shading and coloring can be performed based on consensus rules [7], custom regular expressions [8], and HMMs [9] all selected or defined by the user (Figure 1B-D). We have implemented real-time tracking of cursor position allowing for both column (click-drag) and residue-level (direct edit) control of sequence editing functions without losing positional context or shading information. The SlimShadey GUI is similar to a web browser, allowing several sequence alignments to be opened simultaneously and modified in independent tabs (Figure 1E, new tab).

## Input and output:

SlimShadey accepts nucleic acid or protein sequences in multiple formats such as FASTA (aligned or unaligned), CLUSTAL, or .mm (SlimShadey project format). SlimShadey allows files to simply be dragged and dropped into the GUI, streamlining the input of the alignment. To improve performance with large numbers of sequences, users may collapse identical sequences into a single sequence that represents the group of identical sequences. This is a reversible process that can be performed either before or after rendering. This versatile function allows visualization and performance to be balanced as needed.

Output options include configurable shaded alignments with scalable resolution and sequence logos (.PNG, Figure 1D), alignments in rich text format (.RTF) for use with standard word processors (e.g. Microsoft Word), and FASTA and Clustal formatted alignment files. The SlimShadey project format saves the sequence alignment and all formatting options, allowing alignments with custom shading and edits to be shared across platforms for collaboration.

## Processing:

SlimShadey can align any unaligned sequences or realign edited sequences using Opal [10]. Alignments can be shaded by residue frequency, similarity/identity to a master sequence or consensus, RASMOL coloring [11], ClustalX coloring [12], ScanProsite motifs [8], HMMER domains [9], and common substitution matrices (including PAM and BLOSUM). In addition, Slimshadey is the first to implement shading by unique residues, color gradients based on substitution matrices, and custom regular expressions.

All shading is position independent, ignores gaps, and is redrawn on the base alignment with the user-defined shading unaffected. For example, when shading with ScanProsite motifs, HMMER motifs, and custom regular expressions, SlimShadey identifies motifs, even if gaps have been introduced in the alignment or they occur in different positions in separate sequences. Domains and motifs are initially shown in a "bird's eye" format for the evaluation of gross positional information (Figure 1C).

Cursor tracking on the alignment allows for residue-level control in editing the alignment. Hovering over a character shows its position in its sequence and in the alignment (i.e. counting and ignoring gaps, respectively), as well as its identity, in the lower right of the interface. Clicking on a residue will allow the user to change character identity, character color, and background shading. Clicking on a sequence will allow the user to change sequence name, sequence order, and numbering (i.e. index of the first residue). One of the highlights of SlimShadey is that user-defined subsequences (e.g. N-terminal and C-terminal ends, indels) can be edited, deleted, or directly exported to a new tab for further manipulation (Figure 1E left, new tab). Blocks of sequence can be easily delimited by right-clicking and dragging over the range desired. In addition, once a subsequence is selected, the range of characters can be either shaded as a separate user-defined block, used to generate a sequence logo (Figure 1E right), or annotated with any UTF-8 characters (Figure 1B, annotations). Sequence logos are generated using an information theoretic approach, where a character's height at a position in the logo scales according to its frequency at that position in the alignment [13]. Annotation of sequence alignments can be performed using any valid UTF-8 character, including Greek characters, arrows, and loops to highlight motifs, domains, and other structural features.

## Funding:

This work was partially supported by funding from The College of New Jersey Mentored Undergraduate Summer Experience.
